# Agreement and concordance between married couples regarding family planning utilization and fertility intention in Dukem, Ethiopia

**DOI:** 10.1186/1471-2458-13-903

**Published:** 2013-10-01

**Authors:** Chala W Diro, Mesganaw F Afework

**Affiliations:** 1 Department of Public Health, Faculty of Health Sciences, Wolaita Sodo University, Wolaita Sodo, Ethiopia; 2 School of Public Health, Addis Ababa University, Addis Ababa, Ethiopia

## Abstract

**Background:**

Traditionally, women have been the main respondents for most of knowledge, attitude and practice survey related to family planning and fertility intention. However it is not well known how responses of women about husbands reflect the husband’s attitude and practices. Thus this study is conducted to examine agreement of wife and husband regarding family planning use and fertility desire in Dukem, Ethiopia.

**Methods:**

A community based cross sectional survey was conducted on 422 married couple’s in Dukem town, central Ethiopia which were selected by using systematic sampling method. The study was conducted from November, 2010 to December, 2010. Data pertaining to fertility intentions and contraception was collected and the level of agreement (kappa statistics) between husbands and wives was computed.

**Result:**

The observed concordance was 71.6% for ideal family size, 94.9% for contraceptive attitude, 95.9% for fertility desire, and 99.7% for report of number of currently living children. The unadjusted kappa statistic varied from 0.61(p<=0.000) for contraceptive attitude to high of 0.99(P<=0.000) for number of living children, for ideal family size 0.63(P<=0.000), fertility desire 0.91(P<=0.000), ever use of contraceptive 0.84(P<=0.000) and current use of contraceptive 0.87(P<=0.000) having kappa values in between. Overall greater degree of agreement was observed for reproductive health events as compared to family planning attitudes and intentions.

**Conclusion:**

In surveys pertaining to reproductive health events, the wife’s response may be taken as proxy for the couple’s response, but for assessing family planning attitudes and intentions, may require collecting information from husbands and wives separately.

## Background

Traditionally, women have been respondent for the most of knowledge, attitude and practice survey related to family planning and fertility intentions [1].

However, it has been now realized that programs that exclusively focus on either men or women may fail because most sexual, family planning, and child bearing decision are made or may potentially be able to be made by both partners together and the response by one of them may not reflect the reality [1,2].

Moreover, available studies show that in many developing countries male often dominate when any important decision are taken in the family, such as reproduction, family size, and contraceptive use [3,5-8].

Furthermore, the level of spousal agreement regarding fertility and family planning remains an important area for utilization of reproductive service. Although high concurrence would be expected because of daily partner contact and common living conditions, cross national studies of couple concurrence on contraceptive methods use show frequent discrepancies between husbands and wives reports [4,9-12].

Thus this study examines agreement between wife and husband on family planning use and fertility intention in Dukem central Ethiopia.

## Methods

### Design and study area

Community based Cross-sectional study was conducted in Dukem town, central Ethiopia from November, 2010 to December, 2010. Dukem town is 37 km away to south east of the capital of Addis Ababa and is divided into four kebeles^1^ The town’s total population is 44,009 of which 24,313(55%) are males and 19,696(45%) are females.

### Study population and sampling procedure

The study population comprises married couples aged 15–49 residing in Dukem town that was selected by using systematic random sampling techniques.

Sample size was calculated using single proportion formula as follows;

$$\begin{array}{r} n=\left(Z\alpha /2\right)2\;p\left(1-p\right)\\ d2 \end{array}$$

Where

**n=**the desired sample size.

**p**=proportion of agreement of couples in sub-Saharan countries on contraceptive use 47%-82% take the minimum proportion [13].

Z α/2= critical value at 95% confidence level of certainty (1.96).

**d**= the margin error between the sample and the population =5%.

using the above formula sample size for single population proportion the desired sample size is 383.considering non response rate of 10% the total sample size used for the study was 422 married couples.

**Figure 1:**
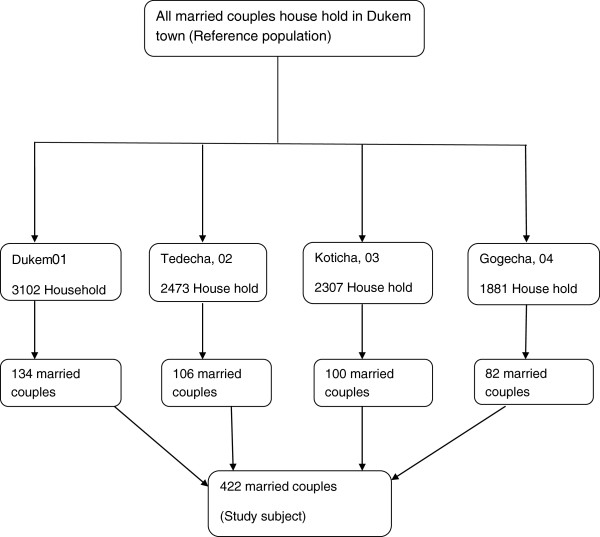
Schematic representation of sampling procedure.

As shown in (Figure 1) the sample was determined proportional to the size of the total house hold population of each kebele. Systematic sampling method was used to select the households from each kebele, where the sampling interval were the total number of households in each kebele divided by the corresponding number of households to be interviewed in each kebele.

The first household interviewed was determined from the kebele house number register using lottery method. The next household was identified by systematically adding the number of interval to the preceding one. If more than one eligible respondent were found in the selected household, only one respondent was chosen by lottery method. In cases where no eligible is identified in the selected household, the interviewer was moving to the next household.

Data were collected using pretested structured questionnaire, which was administered by the trained data collectors. The interviews were conducted at the respondent’s home and both husband and wives were interviewed on the same day but separately (Figure 1).

### Data analysis

Data were entered into Epi info 3.5.1 and analyzed using SPSS 16 soft ware package. First frequencies of the overall distribution of the variables under study were done. Kappa statistics was done to see the level of agreement of couples on family planning utilization and fertility desire.

As observed by others finding an adequate measure for distinguishing between spousal concordance that occurs by chance and actual agreement are methodological difficulty. In addition the Kappa statistics is influenced by the distributions of data across the categories that are used. As Kappa statistics may be influenced by the presence of more than two categories and by high prevalence of the outcome under consideration, we used weighted kappa and prevalence adjusted kappa wherever appropriate [14,15].

### Ethical consideration

Ethical clearance was obtained from the school of public health, and Addis Ababa University College of health science ethical committee. A formal letter was also submitted to all the concerned bodies to obtain their co-operation. Verbal informed consent was obtained from all study participants after explaining the purpose of the study. The participation was voluntarily and they could withdraw from the study at any time without explanation. Confidentiality was assured and no personality identifying details were recorded.

## Result

### Socio demographic characteristics

A total of 422 couples were interviewed. The age range for wives was from 16 to 46 years with mean age of 27.9 and for husband was from 20 to 53 years with mean age of 32.8 years. As indicated in Table 1, majority of men, 383(90.8%) and the women 326(77.2%) reported attending formal education. Most of those who reported attending formal education, 321(76.1%) of men and 297(70.3%) of women attended elementary and high school [grade 10 and below]. About 21% of women and 9% of men were illiterate.

**Table 1:** Socio demographic characteristics of married couples in Dukem town, 2010 (n=422)

		**Wife**		**Husband**	
**Characteristics**		**No**	**%**	**No**	**%**
**Religion**	Orthodox	358	84.8	358	84.8
	Protestant	56	13.3	54	12.8
	Muslim	6	1.4	7	1.7
	Catholic	2	.5	3	.7
	Total	422	100.0	422	100.0
**Ethnicity**	Oromo	292	69.2	289	68.5
	Amhara	120	28.4	112	26.5
	Tigre	2	.5	8	1.9
	Gurage	7	1.7	10	2.4
	Others	1	.2	3	.7
	Total	422	100.0	422	100.0
**Education**	Illiterate	87	20.6	36	8.5
	Read & write	9	2.1	3	.7
	Elementary	120	28.4	78	18.5
	Junior	130	30.8	140	33.2
	High school	47	11.1	103	24.4
	Preparatory	21	5.0	45	10.7
	Higher education	8	1.9	17	4.0
	Total	422	100.0	422	100.0

### Reproductive history

More than half 53.1% of women were married at ages less than 19 years, while only 11.8% men did so. The minimum age at marriage reported for women was 13 years and for men was 15 years. The mean age at marriage was 19.7 and 24.6, and standard deviation 3.9 & 4.7 for women & men respectively.

**Figure 2:**
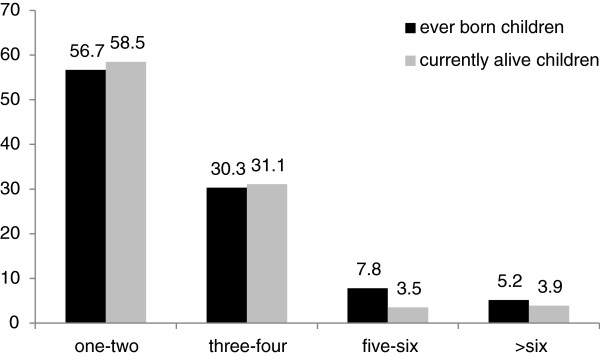
Number of ever born and alive children for married couples in Dukem town, 2010.

386(91.5%) had ever born children and the rest did not have. As shown in Figure 2 Out of this about 56.7% had one to two children, 30.3% had three to four children, 7.8% of them had five to six children, and the rest 5.2% had more than six children ever born and the mean number of ever born children for a couple was 2.7.

Among these ever born children 58.5% one to two children, about 31.1% three-four children, 3.5% five to six children, and 3.9% more than six children respectively were alive children. The mean number of alive children was 2.5 (Figure 2).

**Figure 3:**
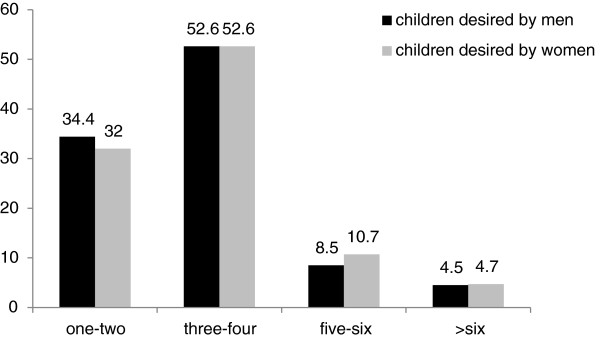
Number of children desired by married couples in Dukem town, 2010(n=422).

About 65.9% of the respondents wanted to have additional children and the rest did not. More than half (52.6%) of a couple wanted to have three to four children, while about one third of couple wanted to have one to two children. The mean of ideal child desire was 3.4 children, where 1.9 was for male child and 1.5 was for female child. The minimum was 1 child and the maximum was 12 children (Figure 3).

### Agreement level between husband and wife regarding contraceptive practice, attitude and fertility desire

Table 2 shows proportional agreement of the number of living children as reported by husbands and wives. Overall in 99.7%(95% CI: 99.2 to 100.2%) of cases, both partners reported the same number of living children and the kappa statistic was 0.99(p<=0.000), which corresponds to excellent agreement. In one case where there was a difference in reported number of children, it was husband who reported more number of children.

**Table 2:** Agreement level between husband and wife regarding contraceptive practice, attitude and fertility desire in Dukem town, 2010/11 (n=422)

**Ever use of contraception**	**Husband**			
**Wife**	**No (%)**	**Yes (%)**	**Total**	**kappa**
No	61(14.5)	3(0.7)	64(15.2)	0.84
Yes	16(3.8)	342(81)	358(84.8)	
Total	77(18.3)	345(81.7)	422(100)	
**Current use of contraceptive**	**No**	**Yes**	**Total**	
No	77(18.2)	6(1.4)	83(19.7)	0.87
Yes	12(2.8)	327(77.5)	339(80.3)	
Total	89(21.1)	333(78.9)	422(100)	
**Contraceptive attitude**	**Disapprove**	**Approve**	**Total**	
Disapprove	19(4.5)	6(1.4)	25(5.9)	0.61
Approve	16(3.8)	381(90.3)	397(94.1)	
Total	35(8.3)	387(91.7)	422(100)	
**Fertility desire**	**Want no more**	**Want more**	**Total**	
Want no more	131(31)	13(3.1)	144(34.1)	0.91
Want more	4(0.9)	274(64.9)	278(65.8)	
Total	135(31.9)	287(68)	422(100)	
**Current living children**				
No	0(0.0)	1(0.3)	1(0.3)	0.99
Yes	0(0.0)	385(99.7)	385(99.7)	
Total	0(0.0)	386(100)	386(100)	
**Ideal family size**				
No	0(0.0)	61(14.5)	61(14.5)	0.63
Yes	59(13.9)	302(71.2)	361(85.5)	
Total	59(13.9)	363(86.0)	422(100)	

As indicated in Table 2, proportional agreement for ever use of contraception irrespective of the method showed 95.5%(95% CI: 94.5% to 96.5%) overall agreement between husbands and wives. The kappa statistic was 0.84(P<=0.000), which corresponds to excellent agreement. Ever use of contraception was reported by sixteen 3.8% wives while husbands reported that they had never used any contraception. In most of the cases, the wives had taken oral pills and it could be that they did not inform their husbands regarding this. Ever use of contraception was reported by three 0.7% husbands when wives reported otherwise. This difference may be attributable to differences of perception between spouses regarding traditional methods or may be indicative of existence of multiple sexual partners condom use.

As shown in Table 2 Current use of contraception as reported by husbands and wives showed 95.7%(95% CI 94.7 to 96.7%) over all agreement. The kappa statistic was 0.87(P<=0.000), which corresponds to excellent agreement. Current use of contraception was reported by twelve 2.8% wives while the husband reported using none. Six 1.4% husbands reported current use of contraception while their wives did not. It may be inferred that there is covert contraceptive use by the couples.

Proportional agreement for fertility desire showed agreement between husband and wives is 95.9%(95% CI: 94.01 to 97.8%) of cases, 31% of couples wanting no more children and 65.9% wanting more children. The kappa statistic was 0.91(P<=0.000). The disagreement was unevenly divided across the remaining two cells, with 4 cases where the wife desired more children while the husband did not and 13 cases where the husband desired more children but wives did not.

Proportional agreement of ideal family size showed that in 71.6%(95% CI: 67 to 76%) of cases there was agreement between husband and wife. The kappa statistics was 0.63(P-value 0.000). In 14.5% of cases, the husband wanted more children than the wife did, while in 13.9% of cases the wife wanted more children than the husband did.

Cross tabulation of attitude towards contraception shows that there was 94.8%(95% CI: 92.8 to 97%) overall agreement between husbands and wives in attitude toward contraception; both partners approved of contraception in 90.3% cases. The unadjusted kappa statistic was 0.6(P<=0.000) which corresponds to good agreement. In 3.8% of cases, wives approved of contraception whereas husbands did not, and in 1.4% of cases, husbands approved of contraception where as wives did not.

## Discussion

This community based study used information from both husband and wife to assess the agreement between married couples regarding family planning utilization and fertility desire in Dukem town.

The age range for wives was from 16 to 46 years with mean age of 27.9 and for husband was from 20 to 53 years with mean of 32.8 years. This mean age difference is due to the fact that only women with age range of 15–49 were included in the study and also mostly women married husband elder than themselves [16-18].

The level of spousal agreement regarding fertility and family planning remains an important area for utilization of reproductive service. For this study on current reproductive attitude and behavior among the couples with both partners in their first marriage only one valid response can be recorded for a couple for objective events like number of children born and current use of contraception. Any differences indicate response error on the part of one spouse or both. Determining which respondent give the correct response is usually impossible because validation studies of these indices are lacking. The minor differences in objective events in the present study can be explained by the male dominated culture of this study population [19-22].

In this study, the observed agreement between husbands and wives regarding reporting of reproductive health events and family planning attitudes and intentions varied from moderate 71.6%(95% CI:67.3 to 75.9) for ideal family size to a high of 99.7%(95% CI:99.2 to 100.2%) for a number of currently living children. But the unadjusted kappa statistic varied from 0.61(P<=0.000) for contraceptive attitude to 0.997(P<=0.000) for a number of living children.

In agreement with this study, research from Turkey reported that spousal reports on most fertility and contraceptive use measures demonstrated moderate to high concordance, where as reports of approval of family planning showed only fair concordance [4,23-26].

This study revealed that high level of agreement in reporting the number of living children between husbands and wives. A high level of agreement in number of living children was also reported by a study done in rural Maharashtra, India where only 0.4% disagreement was observed for number of currently living sons and daughters. They also concluded that women reported live births more accurately than men [27-29].

In this study current use of contraception as reported by husbands and wives showed 95.7%(95% CI 94.7 to 96.7%) over all agreement. The kappa statistic was 0.87(P<=0.000), which corresponds to excellent agreement. In a study using data from six demographic and health surveys of sub Saharan Africa, contraceptive use agreement ranged from 47% to 82%, but among couples in whom one or both reported use, the both category represented less than half in all nations except Zimbabwe [13,30].

Whereas discrepancies in the reporting of events indicate reporting errors on the part of one or both spouses, differences of attitudes and intentions are expected because these are subjective indicators.

Available studies show that in many developing countries, males often dominate in decision making in the family, including in issues related to reproduction, family size and contraceptive use. Research in Kenya suggests that contraception is 2–3 times more likely to be used when husbands, rather than wives, want to cease childbearing [31,32].

Male involvement not only helps in making a contraceptive more acceptable, but also makes its effective use and continuation more likely. On the other hand, even if the wife wants to use a contraceptive, she may not be able to use it or may be forced to discontinue the method if the husband disapproves of contraception. In study conducted in Indonesia, husband’s approval was the most important determinant of contraceptive use [33].

## Conclusion

Overall, a greater degree of agreement was observed for reproductive health events as compared to family planning attitudes and intentions. The latter are more subjective outcomes and can be expected to vary among spouses. Thus, we can infer that for reproductive health events, wives responses can be taken as proxy for the couple’s response but family planning attitudes and intentions may require collection of information both from husbands and wives.

## Endnote

^a^Kebele is the smallest administrative unit in Ethiopia.

## Competing interests

The authors declare that they have no competing interests.

## Authors’ contributions

CW was responsible for the development of study design, implementation, analysis, interpretation and the preparation of the draft manuscript. MF involved in the design, the writing, interpretation and critical revision of the paper for intellectual content. All authors read and approved the final manuscript.

## Pre-publication history

The pre-publication history for this paper can be accessed here:

http://www.biomedcentral.com/1471-2458/13/903/prepub
